# A successful surgical repair for supravalvular aortic stenosis with a bicuspid valve and malpositioned coronary orifices by partial Brom’s technique: a case report

**DOI:** 10.1186/s40792-024-02039-w

**Published:** 2024-10-24

**Authors:** Midori Hara, Yoshihiro Honda, Shigeaki Kaga, Kisaburo Sakamoto, Hiroyuki Nakajima

**Affiliations:** 1 Department of Surgery II, Faculty of Medicine, University of Yamanashi, 1110 Shimokato, Chuo-city, Yamanashi 409-3898 Japan; 2 Department of Cardiovascular Surgery, Mt. Fuji Shizuoka Children’s Hospital, 860 Urushiyama, Aoi-ku, Shizuoka, 420-8660 Japan

**Keywords:** Supravalvular aortic stenosis, Aortic valvuloplasty, Brom’s technique

## Abstract

**Background:**

Supravalvular aortic stenosis (SVAS) is a relatively rare form of left ventricular outflow tract obstruction, often accompanied by other cardiac conditions. However, a standard surgical reparative technique has not been established and repairing SVAS remains challenging.

**Case presentation:**

We repaired SVAS of a 3-year-old boy accompanied by a bicuspid aortic valve and malpositioned coronary orifices by partial Brom’s technique with two glutaraldehyde-treated autologous pericardial patches, using recent advanced preoperative information, including geometric and effective heights. Echocardiography after the surgery revealed release of SVAS without aortic regurgitation.

**Conclusions:**

In repair for SVAS, it is important not only to release stenosis but also to make a functional aortic valve, using recent advanced preoperative information. In the case of children, repairing the aortic valve by only using autologous tissue having growth potential, is also important.

## Background

Supravalvular aortic stenosis (SVAS) is a rare form of left ventricular outflow tract obstruction often accompanied by various complications. Therefore, repairing SVAS remains challenging, with no standard surgical technique. We herein report a case of SVAS accompanied by a bicuspid aortic valve and malpositioned coronary orifices, repaired by the partial Brom’s technique.

## Case presentation

**Fig. 1:**
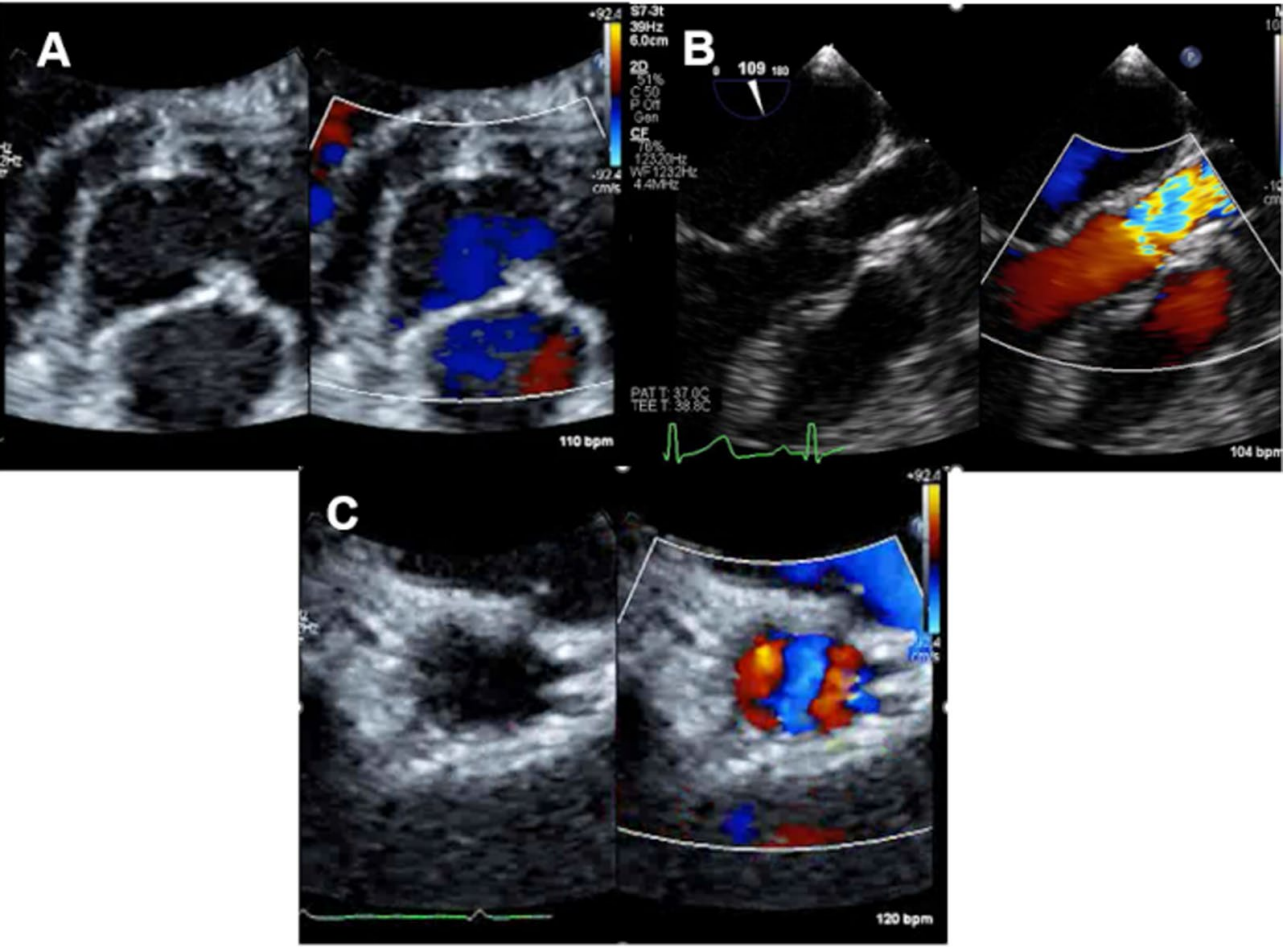
Echocardiographic findings of the aortic valve and the coronary arteries. **A** Echocardiography revealed partial coaptation of the non-coronary cusp (NCC) and right coronary cusp (RCC), resulting in a bicuspid aortic valve. **B** Aortic annulus was 10.3 mm, and the aortic stenosis was 8.0 mm. **C** Both coronary orifices of the left anterior descending artery and the left circumflex artery were located separately in the LCC

**Fig. 2:**
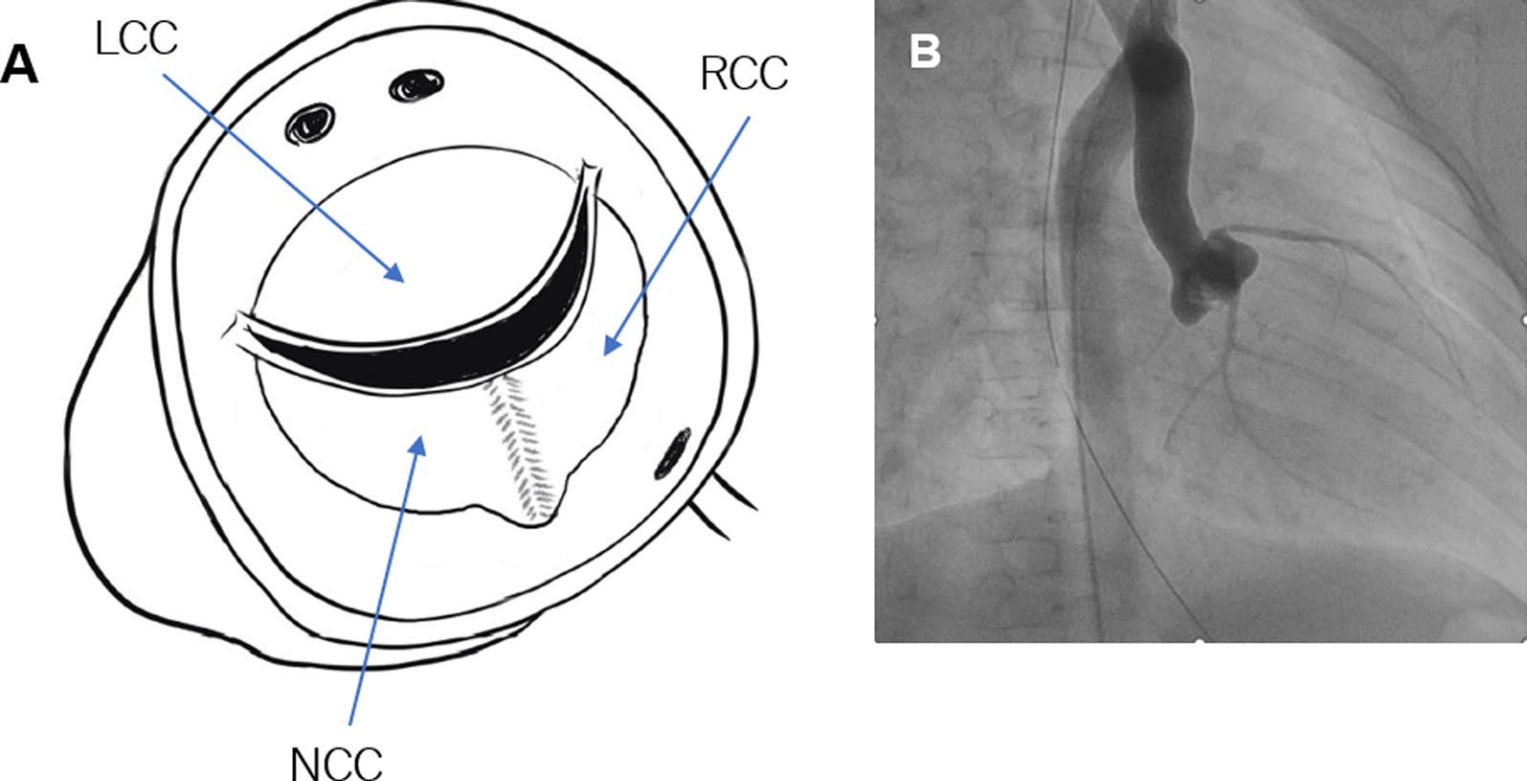
Schematic of the aortic valve and coronary artery orifice, and aortography. **A** Anatomy of aortic valve. **B** Cardiac catheterization revealed a left ventricular systolic pressure of 130 mmHg and ascending aortic pressure of 77 mmHg. Left Ventricle-Aorta pressure gap was 50 mmHg

SVAS was diagnosed in a 2-month-old boy who presented to our hospital with a systolic murmur. At 2 years and 8 months old, the left ventricle–aorta (LV-Ao) pressure gradient measured by cardiac catheterization became 50 mmHg, and a surgery was decided. Echocardiography revealed partial fusion of the non-coronary cusp (NCC) and right coronary cusp (RCC), resulting in a bicuspid aortic valve (Fig. 1A). The aortic annulus was 10.3 mm, and the sinotubular junction was 8.0 mm (Fig. 1B). Both coronary orifices of the left anterior descending artery and the left circumflex artery were separately located in left coronary sinus (Figs. 1C, 2A). To perform aortic valvuloplasty, geometric and effective heights of every cusp were measured by echocardiography as follows: each geometric height of the LCC, RCC and NCC was 13.7, 12.1 and 11.6 mm respectively. Height from the aortic annulus to each cusp was almost the same and every effective height was 6.2 mm. His body weight was 14.6 kg, and body surface area (Du Bois method) was 0.57 m^2^. Cardiac catheterization revealed a left ventricular systolic pressure of 130 mmHg and ascending aortic pressure of 77 mmHg (Fig. 2B). From these data, each cusp was judged to be able to make a symmetrical tricuspid valve by enlarging the NCC and RCC sinuses equally to the LCC sinus.

**Fig. 3:**
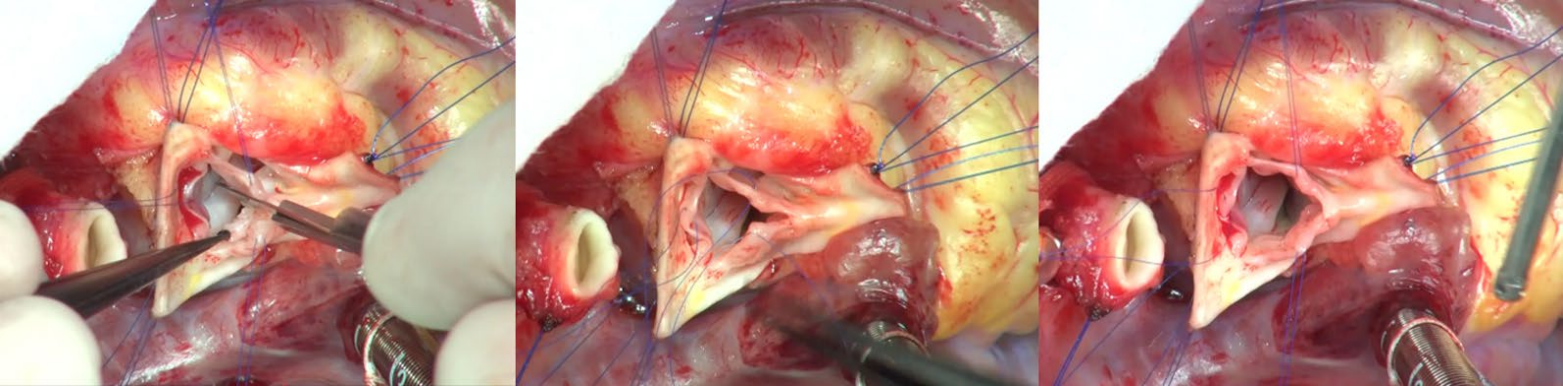
Surgery; aortic valvuloplasty. The adhesed leaflet free margin was incised approximately 4 mm from the aortic annulus to make a tricuspid valve

**Fig. 4:**
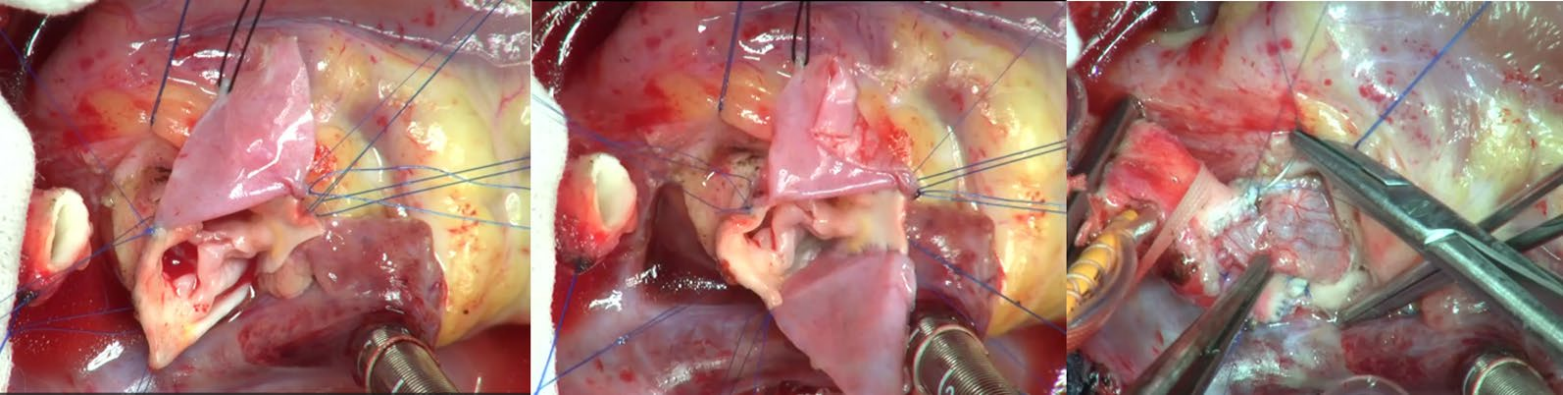
Surgery; enlargement of the aortic root. Glutaraldehyde-treated autologous pericardial patches were sutured into the right and non-coronary sinuses so that we could enlarge the aortic root and make symmetric three sinuses. Then, the ascending aorta was reconstructed

**Fig. 5:**
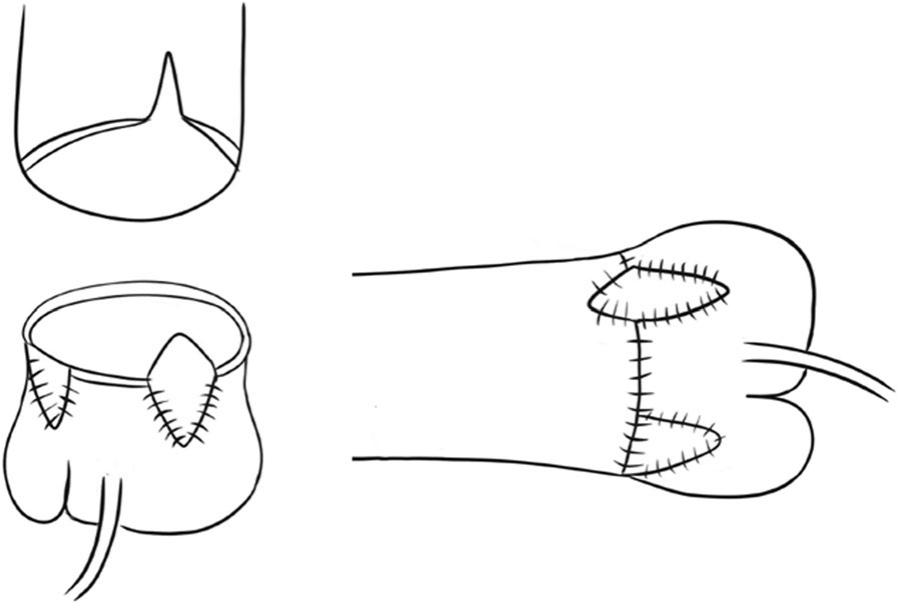
Reconstruction of the aortic root. Distal side of the ascending aorta was enlarged by incisions and insertion of the right sinus’s patch

Standard median sternotomy was performed. After establishing cardiopulmonary bypass and aortic cross-clamping, a transverse incision of the ascending aorta was made approximately 5 mm above the sinus of Valsalva. NCC and RCC sinuses were incised till 2 mm above the aortic annulus. Each geometric height of the LCC, RCC, and NCC measured intraoperatively was 11, 10, and 9 mm, respectively. From these measurements, we reconfirmed that we were able to create a symmetric aortic valve as our presurgical plan. NCC and RCC had a fusion that formed a bicuspid valve. The fused leaflet free margin was incised approximately 4 mm from the aortic annulus because we considered that further incision would have uncontrollable regurgitation (Fig. 3). Since the LCC was large enough and difficult to cut without injuring the two coronary orifices, only the right and non-coronary sinuses were enlarged, using 5-min-glutaraldehyde-treated autologous pericardial patches (Fig. 4). Distal side of the ascending aorta was enlarged by incisions and insertion of the right sinus’s patch (Fig. 5).

Post-operative course was uneventful. Echocardiography on postoperative day 21 revealed a left ventricular tract peak velocity of 2.4 m/s, which was significantly lower than the preoperative velocity of 4.4 m/s. Aortic regurgitation was mild.

## Discussion

Surgical techniques to treat SVAS have developed from simple procedures to complicated reconstructions of the aorta. The earliest technique was McGoon’s single-sinus diamond-shaped patch repair and in 1977, Doty’s 2-sinus inverted bifurcated patch aortoplasty technique was invented. In 1988, Brom started 3-sinus repair, using three patches to reconstruct each aortic sinus. In 1993, Meyers invented an autologous slide-aortoplasty technique. Brom’s 3-sinus repair have gained in popularity because it can repair sinuses flexibly compared with 1- and 2-sinus repairs [1]. It is important not only to release the stenosis but also repairing the sinuses of Valsalva; creating the three sinuses equally will reconstruct more functional valve and contribute to long-term results [2]. Brom’s aortoplasty promotes the restoration of normal aortic root geometry and relief of coronary ostial stenosis, which is important in preventing myocardial ischemia [3].

Our patient had a large enough left sinus with two coronary orifices, and a bicuspid aortic valve, a combination that is exceedingly rare [4]. Though the two sinuses of NCC and RCC were apparently smaller than the LCC sinus (almost normal), the geometric height of the 3 cusps by preoperative echocardiography were acceptable one (11.6–13.7 mm) comparing to the normal (12–14 mm) [5] estimated by body surface area. Making a perfect tricuspid valve is the best. However, we consider it is much more important for young patients to live with their own functioning valve as long as possible. Our major goal is to make an aortic valve, both regurgitation and stenosis controlled within an acceptable level, used only their autologous tissue for each cusp. Thus, we performed valvoplasty without cusp extension and made bicuspid into tricuspid valve. First, we incised the fused leaflet free margin approximately 4 mm from the aortic annulus because further incision would have uncontrollable regurgitation and require commissuroplasty with pericardium patch. If we also did commissuroplasty, the time of the cardiopulmonary bypass would have become longer. Then, we enlarged the non-coronary and right sinuses equally to the left sinus with glutaraldehyde-treated autologous pericardial patches to prevent the left coronary artery from injuring and to create symmetrical sinuses of Valsalva. There is concern that excessive expansion at the sinotubular junction may compromise the coaptation of the aortic valve. Therefore, we designed the width of two patches about 9 mm, considering that the size difference between the diameter of the most stenotic part, 8.0 mm and the normal aortic annulus diameter, 11.0 mm. To enlarge with 2 patches, we took 4.5 mm plus 2 mm for seam allowance, total about 9 mm for each patch. Information including geometric heights and effective heights provided guidance for developing a surgical repair strategy and helped to achieve successful aortic valve repair using two patches.

## Conclusions

In a repair for SVAS, it is important to develop a surgical strategy not only to enlarge the aortic root but also to make a functional aortic valve, using recent advanced preoperative information. Also, to standardize a surgical technique for SVAS, we need accumulating successful cases and analyzing.

## Data Availability

The data that support the findings of this study are available from the corresponding author, YH, upon reasonable request.

## Abbreviations

SVAS: Supravalvular aortic stenosis
RCC: Right coronary cusp
LCC: Left coronary cusp
NCC: Non coronary cusp
